# Othering and Marginalizing the “Queer” in Rwanda: Findings from Public Perceptions and Lived Experiences of LGBT People

**DOI:** 10.1007/s13178-024-01026-y

**Published:** 2024-08-20

**Authors:** K. Stojanovski, E. K. Igonya, L. T Gutabarwa, E. Otukpa, E. Mutuku, B. A. Ushie

**Affiliations:** 1 Department of Social, Behavioral and Population Sciences, Tulane School of Public Health and Tropical Medicine, Tulane University, New Orleans, LA USA; 2 African Population Health Research Center, Nairobi, Kenya; 3 Health Development Initiative, Kigali, Rwanda; 4 Beshi King Development Services, Abuja, Nigeria

**Keywords:** LGBT, Africa, Stigma, Global health

## Abstract

**Introduction:**

The multidimensionality of stigma that LGBT people experience globally necessitates research to explore the processes at work. The study aimed to quantify the level of othering and explore the process of LGBT othering in Rwanda.

**Methods:**

We conducted a sequential cross-sectional mixed-methods study of LGBT lived experiences in Rwanda. We recruited 499 participants to complete the LGBT-specific survey and 1254 for the non-LGBT survey. We conducted 16 in-depth interviews (IDIs) with non-LGBT participants. For LGBT participants, we completed six focus groups with 59 participants, six in-depth interviews, and three digital storytelling interviews. For the quantitative surveys, we conducted multivariable linear regressions and reported beta coefficients and 95% confidence interval estimates examining LGBT discrimination (LGBT survey) and attitudes toward LGBT (non-LGBT survey). We used deductive and inductive thematic and narrative analysis to analyze the qualitative data.

**Results:**

In adjusted analyses of the non-LGBT survey, as compared to those who knew zero LGBT persons, persons who knew more than five had lower negative attitude scores (β =  − 1.3, 95% CI − 2.2, − 0.5), while the score was lower for those that knew one to five (− 0.2), it was not significant (95% CI − 0.8, 0.5). In the LGBT survey, adjusted analyses indicated that there was no significant difference found in discrimination between bisexual, gay, or lesbian participants. However, as compared to cisgender participants, transgender participants had discrimination scores that were 2.1 points higher (95% CI 1.1, 3.0), and gender non-confirming individuals had scores that were one point higher (95% CI 0.2, 1.9). The qualitative findings showcased how societal “othering” occurs in the everyday life of LGBT Rwandans, with large ramifications in creating feelings of isolation and hampering one’s capacity to live authentically and with dignity.

**Conclusion:**

The findings from our study indicate a high level of othering of the LGBT community across multiple domains, including housing, employment, healthcare, education, religion, and family within Rwanda.

**Policy Implications:**

The findings highlight the importance of social education campaigns about LGBT people, particularly among vital societal role-holders, including healthcare providers and educators. Integrating the rich historical and indigenous culture related to LGBT could be successful in combating anti-West rhetoric.

## Introduction

Worldwide, people who identify as lesbian, gay, bisexual, and transgender (LGBT) face stigma that socially excludes them by categorizing them as “others” or “abnormal” in society. For LGBT people, sexual orientation, gender identity, and behaviors are intersectional elements engendering their othering. The othering of LGBT has severe health and social harms that create inequities between LGBT and non-LGBT people. Such harms include increased suicidality, depression, adverse coping (e.g., smoking), worse sexual health and physical health outcomes, and negative economic and housing impacts (Bränström & Pachankis, 2022; H. Brooks et al., 2018; V. Brooks, 1981; Meyer, 2003; Restar et al., 2020; Russell & Fish, 2016; Saraff et al., 2022; Sherman et al., 2020; Stojanovski et al., 2022; Stojanovski et al., 2018; White Hughto et al., 2015).

Erving Goffman, a prominent stigma scholar, conceives social exclusion as unfolding when “society establishes the means of categorizing persons and the complement of attributes felt to be ordinary and natural for members of each of these categories,” otherwise known as “othering” (Goffman, 1963). Othering is a sociological process in which individuals or groups are classified as unusual from what is considered “normal” because of their social identities. Othering creates differential (stigmatized) treatment or marginalization of the “othered” (i.e., LGBT people) across levels of the socio-ecological model that serves, ultimately, to promote the social exclusion of specific individuals from society (Bronfenbrenner, 1974; Brons, 2015). Othering operates within four key premises (Goffman, 1963). First, othering is predicated on power, privilege, and the ability to shape access to and use of tangible and intangible resources. Second, othering can only arise and be enacted through social relationships and interactions (i.e., there must be an in-group and out-group). Third, it is shaped by cultural norms and values within a place and time. Finally, othering exists on a spectrum, which can be less severe (e.g., interpersonal discrimination) or extremely severe (e.g., criminalizing laws). Consequently, othering is a fundamental cause of inequities because it shapes who can access and leverage their social, political, and human rights (Hatzenbuehler et al., 2013; Link & Phelan, 1995). As the diversity of global literature portrays, the othering of LGBT people and its implications are widespread.

In Africa, the othering of LGBT people is prevalent and operates at the individual, interpersonal, community, institutional, and structural levels. For example, anti-same-sex rhetoric among some African leaders (individual level) can influence public perceptions of the LGBT community (community level) and has the power to shape public policies (structural level). However, these relationships are not unidirectional; for example, laws and court decisions can improve community perceptions about and interpersonal relationships with LGBT people (Tankard & Paluck, 2017). At the structural level, criminalization of same-sex sexual relationships remains illegal in 30 out of 54 African countries (International Lesbian and Gay Association, 2017-2024). Many of the anti-LGBT laws (i.e., anti-sodomy laws) were introduced by Western imperial nations during colonialism and still have effects on LGBT rights in contemporary Africa (Kalende, 2023). In November 2021, a Botswana Appeals Court upheld a lower court’s ruling that banning same-gender consensual relationships was unconstitutional (Reid, 2022). In Angola, legal changes in 2021 saw the decriminalization of same-gender conduct and anti-discrimination legislation (Reid, 2022). While progress has been made in recent years, backlash against LGBT rights and the community is unfolding. For example, in 2023, Uganda passed one of the most draconian laws where LGBT people face long prison sentences and, in some cases, the death penalty for “aggravated homosexuality” (Nicholls & Princewill, 2023). The state of LGBT people’s rights and freedoms is still precarious even in countries with legal status; in South Africa, one of the few countries where same-sex relationships, including marriages, are legal, murders and incidences of corrective rape targeting lesbians have been reported (Koraan & Geduld, 2015; Morrissey, 2013). Other acts of social exclusion include interference with the privacy of individuals, restrictions on the freedoms of assembly, violence, discrimination in and denial of healthcare, education, employment, housing, and arbitrary arrest by law enforcement (Altman et al., 2012; Fay et al., 2011; Francis et al., 2019; Kokogho et al., 2021; Moyer & Igonya, 2018; Müller, 2017; Müller et al., 2021; Zahn et al., 2016).

Rwanda is one of the few African countries with progressive laws and policies. Rwanda has ratified international and regional agreements protecting LGBT people’s human rights and endorsed the United Nations resolution condemning violence against LGBT people in 2011 (UN Human Rights Council, 2015). The 2015 Constitution of Rwanda protects all citizens against discrimination based on any form of difference (The Constitution of the Republic of Rwanda, 2015). Article 16 of the Constitution indicates that all Rwandans are born and remain equal regarding rights and freedom from discrimination. Decades-long civil society advocacy efforts culminated in the 2010 revised penal code, which completely removed articles criminalizing same-sex relationships (Paszat, 2022a). Despite the legal frameworks, the political leadership portrays being LGBT as un-Rwandan, and harassment, stigma, and exclusion are still regular occurrences (Igonya, 2022). While LGBT organizing in Rwanda is active, it is rooted in respectability politics—a political strategy wherein marginalized communities hide and abandon aspects of their sociocultural identity to assimilate, not provoke negative attention, and appear acceptable to the broader society and government (Paszat, 2022b).

The othering has harmed the health of LGBT people in Rwanda. A 2019 study in Kigali among men who have sex with men (MSM) indicated that social stigma isolates them from family, friends, and the larger community and creates fear of accessing services (Adedimeji et al., 2019). Moreover, social stigma was associated with sexual behaviors that elevated MSM’s risk for HIV (Adedimeji et al., 2019). The defining of LGBT as a “key population” itself may lead to social exclusion given “preferential” treatment (e.g., the consideration of LGBT people as a diseased group) or focus within programming and policies.

The multifaceted process of othering that LGBT people experience globally, particularly in Africa, necessitates research to explore the processes at play and understand what drives othering and the experiences of the othered (i.e., LGBT people). This study aimed to (1) quantify the level of othering of LGBT people in Rwanda, (2) assess the factors associated with othering, (3) identify LGBT groups that experience othering, and (4) examine how LGBT people in Rwanda experience othering and in what domains of life.

## Methods and Materials

### Study Setting

**Fig. 1:**
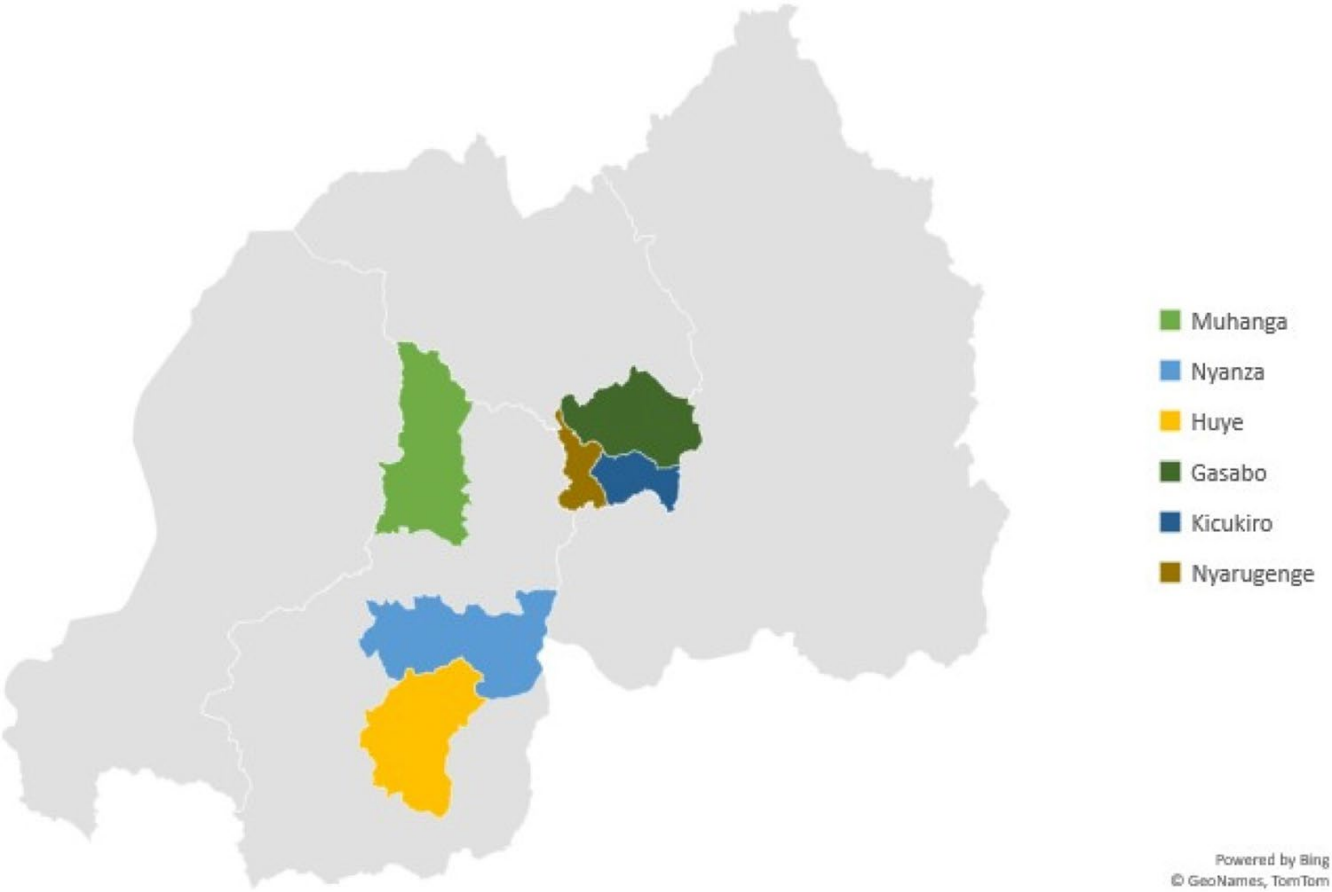
Geographic locations of data collection in Rwanda

We conducted a sequential mixed-methods cross-sectional study in Rwanda, collecting quantitative data first and then using qualitative data to provide depth, context, and nuance (Fetters et al., 2013). We conducted the study in six districts: Gasabo, Kicukiro, and Nyarugenge districts in the Kigali City province and the districts of Muhanga, Nyanza, and Huye in the Southern Province (Fig. 1). We chose these regions because of their significance to Rwandan society (e.g., the Southern Province is the center of Rwandan culture, Nyanza is the King’s Palace Museum, Kigali is the capital, and Huye is home to the University of Rwanda).

### Study Design, Sampling, and Study Populations

Study participants were 18 years or older, could answer questions in English or Kinyarwanda, and had lived in Rwanda for over 6 months. We had two distinct samples: (1) non-LGBT people (*n* = 1254) and (2) LGBT people (*n* = 499). We utilized two forms of data collection: quantitative surveys with both samples and qualitative methods, including focus group discussions and in-depth interviews (IDIs). Before field implementation, we trained specially selected research assistants experienced in working with the LGBT community in various aspects of research ethics, quantitative data collection, qualitative interview techniques, values clarification, and attitude transformation to improve their knowledge and sensitize them about LGBT issues. The quantitative survey data was collected electronically using the SurveyCTO platform, facilitating real-time review and quality checks. We piloted the survey in one southern district.

#### Non-LGBT Sampling and Design

For the survey with non-LGBT people, the chosen parameters for the power analysis were informed by prior literature on similar topics (Anyamele et al., 2005; Bercaw, 2022; Chapman et al., 2011). While discrimination exists against LGBT people in Rwanda, the Rwandan population in contact with the LGBT community is limited, and thus, we use small effect sizes. We used G*Power software (Faul et al., 2009) to estimate the sample size for this study to investigate discrimination and violence enactment against LGBT people. Based on a small effect size (Cohen’s *d* = 0.5), a significance level (α) of 0.05, a desired power (1 − β) of 0.80, and independent *t*-tests of mean differences in perceptions and attitude scores between discriminatory and non-discriminatory groups, we calculated a sample size of 1110 participants adjusted for incomplete responses. We employed convenience sampling to recruit participants for the survey by mapping places where people congregate in large numbers, such as markets, construction sites, bus stations, and places of worship for recruitment. We contacted 1256 people, of which 1254 agreed to participate and completed the survey.

For the qualitative portion with non-LGBT people, we conducted key informant interviews (KIIs) with key stakeholders who have an influence on the daily lived experiences of LGBT people in Rwanda. We included police officers, small business owners, healthcare providers, religious leaders, and civil servants. We used purposive sampling to select key informants based on their sphere of influence on the LGBT community. We contacted 27 individuals for the informant interviews, of which 13 completed the interviews. However, we could not interview some critical individuals from government ministries, law enforcement, and district authorities because they declined participation in this “sensitive” topic. These interviews aimed to gather additional nuanced and contextual information about how the “public” views the LGBT community, such as social attitudes, interactions with LGBT, and awareness of LGBT-related politics and policies. After the quantitative data was collected and analyzed, interviews were conducted at participant offices, churches, or health clinics. No stipends were provided for non-LGBT participants.

#### LGBT Sample and Design

For the survey with LGBT people, an unknown population in Rwanda, we calculated a minimum sample using formulas for an unknown population as follows: *n* = (*Z*-score)^2^ * *p* * (1 − *p*)/*m*^2^ * deff, where *n* = desired sample size, *Z*-score = 1.96, *p* is the estimated population proportion (0.5), *m* is the margin of error (0.5), and deff is the design effect (1.3) (Shete, 2020). We used a 95% confidence interval and a design effect of 1.3 to reduce the loss of precision due to clustering, and the estimated sample was 495 LGBT participants. We adopted a respondent-driven sampling (RDS) approach to reach LGBT people, which suits “hard-to-reach populations,” especially those experiencing stigma (Heckathorn, 1997). We initially identified four seed respondents through LGBT community-based organizations (CBOs) in the Health Development Initiative’s (HDI) network in each surveyed district. We selected seeds depending on their social connections and status within the LGBT community. We gave the seeds six coupons to refer peers. Each study coupon had a unique, non-replicable recruitment number to identify peer networks that fit the inclusion criteria and were color-coded to the specific regions. The process of coupon management was not specific to each LGBT group. Each group, across their personal networks, recruited other identity groups, portraying the interconnectedness of the populations. In addition, 47% of the LGBT sampled were gay, bisexual, or other men who have sex with men (GBMSM), indicating the tighter network relationships within that network. We contacted 502 LGBT persons, of which 499 agreed to participate, consented, and completed the survey. Participants received the equivalent of $10 for transport reimbursement as they met interviewers at a location different from their usual places of residence.

For the qualitative portion of the LGBT sample, we conducted focus group discussions (FGDs) and in-depth interviews (IDIs). We used purposive snowball sampling to identify LGBT people for IDIs (for those with privacy concerns) and FGDs. Data saturation (i.e., uncovering repetitive themes, ideas, and opinions) determined the number of participants. We conducted five FGDs with 50 LGBT participants and five IDIs. Research assistants took notes and documented non-verbal cues to provide context for transcripts. The IDIs were mainly individuals of higher socioeconomic status who had not come out as being LGBT. The IDIs and FGDs were conducted in Kinyarwanda at NGO offices, with participants offered the equivalent of $10 for a transport refund.

### Data Collection Instruments

#### Non-LGBT Data Collection

In the non-LGBT sample, we used a questionnaire exploring societal attitudes toward the LGBT community. For this study, we focused on public attitudes and perceptions toward LGBT people. Specifically, within parts of the survey, we asked about discrimination in different areas of society, such as workplaces and places of worship. The questionnaire was interviewer-administered (face-to-face) at the convenience of the participants. Field interviewers located spaces such as schools, churches, and hotels at various locales to conduct the interviews.

We used semi-structured interview guides for the KIIs with non-LGBT participants’ insights on perceptions and attitudes toward LGBT people in Rwanda. Based on survey findings, we incorporated additional questions (such as who, including institutions, are discriminating against LGBT individuals) into the interview guides to enhance the mixed-methods approach to the study. We gathered information about interactions with LGBT people, personal attitudes and beliefs about the LGBT community, and perceptions of individual and societal treatment of LGBT people. The interviews were digitally audio-recorded, transcribed in Kinyarwanda, and translated into English.

#### LGBT Data Collection

We also used a questionnaire among LGBT participants to gather insights into their lived experiences instrument. We used the Survey on the Lived Experiences of the LGBT + Community questionnaire initially used in South Africa with adaptations to address important linguistic variation in Kinyarwanda (Buntse & Swanepoel, 2020). The survey domains included demographic characteristics, views, and experiences on the political and legal environment, nature and magnitude of discrimination, public tolerance, and family dynamics. Additionally, the survey explored discrimination in access to essential services, unfair treatment at work, eviction from places of residence, denial of rights to participate in social and religious events, harassment, abuse, economic security, and mental health issues.

For the qualitative data collection, we developed and used interview guides to assess cultural and societal attitudes toward LGBT people, knowledge of legal protections against discrimination, lived experiences in everyday life, and their service and program needs. We performed a preliminary analysis of the quantitative data to identify gaps for further exploration in the qualitative component. The FGDs and IDIs were digitally audio-recorded, transcribed in Kinyarwanda, and translated into English.

### Data Analysis

We utilized a mixed-methods explanatory sequential approach to analyze the data across the various sources (Fetters et al., 2013). While we explain our analyses using the sample and data collection method, the analysis was triangulated across data sources. First, we examined specific variables within each survey that measured “othering” and triangulated them across the qualitative data as described in the qualitative analysis section. The quantitative data were analyzed in Stata 15.1 and the qualitative data in NVivo version 10 (*NVivo (Version 10)*, 2014; StataCorp, 2015, p. 15).

#### Non-LGBT Measures and Analysis

For the dependent variable in the non-LGBT survey, we utilized the LGBT acceptance variable. We created a continuous variable from four questions that measured personal attitudes toward same-gender-loving people. The questions had a Likert scale of 1–5, from strongly disagree (1) to strongly agree (5). The four statements were as follows: (1) I feel positive toward LGBT people, (2) LGBT people are mentally sick, (3) I support LGBT rights, and (4) I believe that LGBT people should be treated like any other person under the law. We reverse-scored the responses so that higher scores indicate more discrimination toward LGBT people, and the possible range was four to 20. The question was adapted from the Progressive Prudes Survey by adding questions (3) and (4) above. For the independent variable, we used a variable that measured social interaction and closeness to LGBT people. The question asked how many LGBT people participants knew and were categorized as zero (0), one to five (1), and more than five LGBT people (2). We also included age, sex assigned at birth, educational attainment, employment status, and religious affiliation as control variables. Participants indicated their age in years. Sex was measured by biological sex assigned at birth: male or female. Gender identity was more expansive, measuring self-identified gender (cisgender, transgender, queer). The highest education attained was categorized as primary or lower, post-primary or secondary, and post-secondary. Participants’ occupational status was indicated using the options employed or unemployed. We collapsed religious affiliation to Christianity, Islam, and Traditionalist/no religion.

We used descriptive statistics (frequencies, percentages, means, and standard deviations) to summarize the dependent (LGBT support) and independent variables (known LGBT people), as well as age, sex at birth, religion, education, and occupation variables reached by the participants. Secondly, we conducted bivariate analyses of the sociodemographic characteristics and views on same-sex relationships to explore variation across the sample. Lastly, we conducted multivariable linear regression models to explore the associations between LGBT support and knowing LGBT people and controlled for important confounding variables (e.g., education, employment) from the bivariate analyses. We report beta coefficients and 95% confidence intervals of the regression estimates.

#### LGBT Measures and Analysis

For the dependent variables of the LGBT survey, we utilized the Experiences of Discrimination Scale, which has been widely studied globally (Krieger et al., 2005). We created a continuous variable from thirteen questions (yes vs. no) that measured discrimination due to sexual orientation and gender identity across different life contexts (e.g., work, education, social media, housing, healthcare services). The total possible range was zero to 13, with higher scores indicating more experiences of discrimination. For the independent variables, we created two categorical variables: (1) sexual orientation, defined as gay, lesbian, and bisexual, and (2) gender identity, defined as cisgender, transgender, non-conforming, and genderless. For the sexual orientation analysis, we dropped 41 observations because they identified as straight but not a gender minority. Another 67 responded as straight but identified as a gender minority and were included in the sample. We included age, sex assigned at birth, educational attainment, and employment status as control variables. Participants indicated their age in years. Sex was measured by biological sex assigned at birth: male or female. The highest education attained was categorized as primary or lower, post-primary or secondary, and post-secondary. Participants’ occupational status was indicated using the options employed or unemployed.

We used descriptive statistics (frequencies, percentages, means, and standard deviations) to summarize the dependent (discrimination) and independent variables (sexual orientation and gender identity), as well as age, sex at birth, religion, education, and occupation variables reached by the participants. Secondly, we conducted bivariate analyses of the independent and sociodemographic variables and the discrimination outcome. Lastly, we conducted multivariable linear regression models to examine the associations between discrimination and sexual orientation and gender identity (two separate models) while controlling for important confounding variables (e.g., education, employment) from the bivariate analyses. For the sexual orientation model, we removed the straight-identifying participants, given they identified as a gender minority. We report beta coefficients and 95% confidence intervals of the regression estimates.

#### Qualitative Data Analysis

All the qualitative data was transcribed in the local language, taking into consideration important linguistic and cultural nuances for interpretation. Transcripts were then translated into English for analysis. To analyze the data, we used deductive and inductive thematic and narrative analysis (to assess the dialogic process among focus group participants), combining the qualitative data across the two samples (Reissman, 2008; Vaismoradi et al., 2013). Two researchers and a professional coder iteratively developed a coding framework. Deductively, we were guided by the othering framework and its four major premises examining how othering of LGBT persons in Rwanda (1) is predicated on power and privilege, (2) is enacted through social interactions, (3) is shaped by social and cultural norms, and (4) exists as a spectrum. Inductively, we identified and expanded the major themes and narratives to provide nuance and context to how the theme is experienced and unfolds. Notes taken during interviews and discussions ensured important sociolinguistic nuance during analysis.

## Ethical Considerations

The Rwanda National Ethics Committee approved the study (No. 117/RNEC/2021). The APHRC Internal Ethics Review Committee reviewed and approved the study protocol before submission to the Rwanda National Ethics Committee. All persons who participated in the study gave informed consent. The study team anonymized all data, assigning participants unique identifiers to protect their identities.

## Results

The results are integrated using both the quantitative and qualitative data and were thematically categorized into the following: (1) LGBT othering and its predictors, (2) social isolation as an outcome of othering, and (3) hampered services and capacity to live as an outcome of othering.

### Non-LGBT Sociodemographic Characteristics

A total of 1254 non-LGBT participants completed the survey. Among the non-LGBT sample, 47% (*n* = 584) were 26–35 years of age, and the second largest group was 18–25 years at 27% (*n* = 332). Of the sample, 61% (*n* = 762) identified as male at birth, 62% had a secondary education (*n* = 697), and 65% were employed (*n* = 820). Regarding religion, 82% (*n* = 1000) stated they were Christian. Most participants did not know any LGBT people, 46% (*n* = 549), 39% (*n* = 467) knew one to five LGBT people, and 15% (*n* = 180) knew more than five LGBT people (Table 1).

**Table 1:** Sociodemographic characteristics of non-LGBT participants

Variable	Number (%)
Age groups (*n* = 1254)	
18–25	332 (26.5%)
26–35	584 (46.6%)
36–45	239 (19.1%)
46–45	70 (5.6%)
56–65	26 (2.1%)
> 65	3 (0.2%)
Sex at birth (*n* = 1241)	
Male	762 (61.4%)
Female	479 (38.6%)
Education level completed (*n* = 1123)	
Primary/pre-primary	321 (28.6%)
Secondary/vocational	697 (62.1%)
Post-secondary/Tertiary	105 (9.3%)
Occupation status (*n* = 1254)	
Unemployed	434 (34.6%)
Employed	820 (65.4%)
Religion (*n* = 1222)	
Christianity	1,000 (81.8%)
Islam	121 (9.9%)
Traditionalist/no religion	101 (8.3%)
Number of LGBT people I know (*n* = 1196)	
None	549 (45.9%)
One to five	467 (39.1%)
More than five	180 (15.1%)

### LGBT Sociodemographic Characteristics

A total of 499 LGBT people participated. Among the LGBT sample, 47% (*n* = 233) stated they identified as gay, 16% (*n* = 79) identified as lesbian, 24% (*n* = 119) as bisexual, and 13% (*n* = 67) as heterosexual (all identified as transgender or non-conforming) (Table 2). Examining gender identity, 34% stated they were cisgender males (*n* = 170), 13% (*n* = 64) as cisgender females, 19% (*n* = 94) stated agender, 14% (*n* = 72) listed non-conforming, 7% (*n* = 35) stated they were transgender female, another 7% stated transgender male, and 6% (*n* = 28) were gender fluid (Table 2). Additional demographics are in Table 2.

**Table 2:** Sociodemographic characteristics of LGBT participants

Variable	Number (%)
Age groups (*n* = 499)	
18–25	269 (53.9%)
26–35	196 (39.3%)
36–45	232 (6.4%)
46–45	2 (0.4%)
Sex at birth (*n* = 484)	
Male	139 (26.7%)
Female	355 (73.3%)
Education level completed (*n* = 478)	
Primary/pre-primary	132 (27.6%)
Secondary/vocational	323 (67.6%)
Post-secondary/tertiary	23 (4.8%)
Occupation status (*n* = 474)	
Unemployed	322 (67.9%)
Employed	152 (32.1%)
Sexual orientation (*n* = 499)	
Asexual	1 (0.2%)
Bisexual	119 (23.9%)
Gay	233 (46.7%)
Lesbian	79 (15.8%)
Heterosexual (all gender minorities)	67 (13.4%)
Gender identity (*n* = 499)	
Cisgender male	170 (34.1%)
Cisgender female	64 (12.8%)
Transgender female	35 (7.0%)
Transgender male	36 (7.2%)
Gender fluid	28 (5.6%)
Agender	94 (18.8%)
Non-conforming	72 (14.4%)

### LGBT Othering and Its Predictors

Among the non-LGBT sample, quantitative analysis shows the average score for LGBT support was 11.99 (standard deviation = 4.7), indicating overall negative attitudes toward the LGBT community. There was variation in the score, such that scores were lower among respondents who knew more about LGBT people. From the ANOVA, respondents who knew no LGBT people had a score of 12.5; among participants who knew one to five, it was 11.9; and 10.5 among those who knew more than five LGBT persons (data not shown). The scores were statistically different between respondents who knew more than five LGBT people, as compared to none (*p* < 0.000) and one to five (*p* < 0.001), but not between those who knew one to five LGBT people compared to none (*p* < 0.209, *F* = 12.7). These relationships remained in adjusted regression models. As compared to those who knew zero LGBT persons, persons who knew more than five had scores that were 1.3 points lower ((95% CI − 2.2, − 0.5), *t* =  − 3.02), while the score was lower for those who knew one to five (− 0.2), it was not significant ((95% CI − 0.8, 0.5), *t* =  − 0.48) (Table 3).

**Table 3:** Adjusted beta coefficients between knowing/closeness to LGBT persons and discrimination among non-LGBT respondents (*n* = 1029)

Variable	β	95% CI	*T*-test statistic
# of LGBT known			
None	ref	–	
One to five	− 0.15	(− 0.77, 0.47)	− 0.48
More than five	− 1.31	(− 2.16, − 0.46)	− 3.02
Sex at birth			
Male	ref	–	
Female	− 0.30	(− 0.86, 0.27)	− 1.02
Occupation			
Unemployed	ref	–	
Employed	0.80	(0.19, 1.41)	2.57
Education level completed			
Primary or less	ref	–	
Secondary/vocational	-1.17	(− 1.83, − 0.52)	− 3.52
Post-secondary/tertiary	− 1.43	(− 2.52, − 0.34)	− 2.58
Age			
18–25	ref	–	
26–35	0.37	(− 0.31, 1.06)	1.07
36–45	1.79	(0.93, 2.66)	4.07
46–55	1.67	(0.32, 3.02)	2.43
56 +	2.36	(0.42, 4.29)	2.39
Religion			
Christian	ref	–	
Islam	− 0.15	(− 1.11, 0.82)	− 0.30
Traditionalist	− 0.49	(− 1.58, 0.59)	− 0.90

The qualitative data supported the quantitative findings on negative attitudes toward LGBT individuals. Participants in key informant interviews shared society’s negative attitudes toward LGBT people in Rwanda, “Everything revolves around the way they [LGBT] are perceived in the communities they live in. For instance, the way they’re denied different services, the way people talk bad things behind their backs and generally discriminate against them in conversations and other social activities, and how they’re not welcomed in the society” (KII, Nurse, Huye). The qualitative findings also suggest that religion plays a tremendous role in othering, given its view that being LGBT is a sin and a choice. This view was conveyed in an interview in the following way: “I will not tell you what you want to hear (laughs casually). I would say that the LGBT people and their choices are not the problem; the sinful nature is the problem, and none of us brought it upon ourselves, but we do have the responsibility to choose to come out of the sinful nature by making the right choices” (Muhanga, Pastor, KII).

Experiences of discrimination among LGBT participants were prevalent across the LGBT spectrum. The results indicated that about 67% of LGBT respondents had experienced some form of discrimination. The average discrimination score was 2.95 (out of 13, SD = 3.5, range 0, 13). The ANOVA results indicated elevated discrimination among non-cisgender participants (*F* = 8.59). As compared to cisgender participants (mean = 2.32), transgender participants had discrimination scores that were 2.3 points higher (*p* < 0.000), non-conforming participants scored 1.2 points higher (*p* < 0.034), and agender participants were not statistically significant (data not shown). In adjusted regression models of sexual orientation and discrimination, there was no significant difference in discrimination between bisexual, gay, or lesbian participants (Table 4). In adjusted regression models between gender identity and discrimination, we found variation across identity groups. Compared to cisgender participants, participants who were transgender had discrimination scores that were 2.1 points higher ((95% CI 1.1, 3.0) *t* = 4.10), and gender non-conforming participants had scores that were one point higher ((95% CI 0.2, 1.9), *t* = 2.29) (Table 5).

**Table 4:** Adjusted beta coefficients between sexual orientation and experiences of discrimination (*n* = 374)*

Variable	β	95% CI	*T*-test statistic
Sexual orientation			
Bisexual	ref	–	
Gay	0.74	(− 0.73, 2.20)	0.99
Lesbian	0.26	(− 0.69, 1.20)	0.54
Sex at birth			
Female	ref	–	
Male	0.50	(− 0.97, 1.98)	0.67
Occupation			
Employed	ref	–	
Unemployed	0.85	(0.10, 1.61)	2.21
Education level completed			
Primary or less	ref	–	
Secondary/vocational	0.54	(− 0.62, 1.70)	0.92
Post-secondary/tertiary	1.61	(0.80, 2.43)	3.89
Age			
18–25	ref	–	
26–35	0.70	(− 0.03, 1.42)	1.88
36–45	1.79	(− 0.05, 2.80)	1.90
46–55	0.49	(− 7.25, 6.27)	− 0.14

^*^Analysis excludes straight-identifying participants as they identified as transgender and only includes participants with responses across all variables

**Table 5:** Adjusted beta coefficients and 95% confidence intervals between gender identity and experiences of discrimination (*n* = 413)*

Variable	β	95% CI	*T*-test statistic
Sexual orientation			
Cisgender	ref	–	
Transgender	2.05	(1.07, 3.03)	4.10
Non-conforming	1.03	(0.15, 1.92)	2.29
Agender	0.77	(− 0.09, 1.63)	1.76
Sex at birth			
Female	ref	–	
Male	0.52	(− 0.24, 1.27)	1.35
Occupation			
Employed	ref	–	
Unemployed	0.71	(0.003, 1.43)	1.97
Education			
Primary or less	ref	–	
Secondary/vocational	0.60	(− 0.51, 1.71)	1.06
Post-secondary/tertiary	1.68	(0.92, 2.44)	4.35
Age			
18–25	ref	–	
26–35	0.50	(− 0.18, 1.18)	1.44
36–45	0.82	(− 0.55, 2.19)	1.18
46–55	0.03	(− 4.67, 4.73)	0.01

^*^Analysis includes participants with responses across all variables included in the model

Qualitative data corroborates quantitative data, revealing negative lived experiences and stigma, especially among LGBT people who do not conform to typical “gender norms” and experience worse discrimination. For instance, a participant said a transgender may attract more attention than a gay based on their appearance and stated, “The most affected among us are the trans [people]. When you happen to meet up any authorities, they make fun of you. they call people on you, make fun of you, where you feel embarrassed.” (Kigali, R4, FGD). Others have suggested that “feminine” mannerisms among gay men and the dressing of transgender participants are most othered as they do not conform to the typical gendered cultural norms, as a gay man described being othered:

“...in most cases, you cannot tell who is gay simply by their physical appearance. But there are those others who are not that lucky because they have feminine features and gait naturally, so I do not mean transgender. People like those face more challenges because they get rejected 100% and can’t camouflage; whether they try to hide it or not, they’ll be seen. I have many friends like that. Even though they are my friends, I can be ashamed to invite them to hang out with my straight crew because I’m afraid that if they show up, it’ll be obvious to everyone that I’m gay, you see!” (Kigali, VIP Sales Associate, IDI).

Nevertheless, another participant told us all LGBT groups are stigmatized, “From my point of view, I don’t see any difference. They treat everyone the same, either gay, lesbian, trans and others in the LGBT community, so there is no difference in the way society treats us” (Kigali, R5, FGD).

### Social Isolation Because of “Othering”

Societal “othering” has far-reaching ramifications in creating feelings of isolation among the LGBT community in Rwanda. The isolation occurs in many areas of life, particularly within families. As a participant in an IDI shared their experiences of being LGBT and family relations, “Another challenge is when your family becomes aware of whom you are [LGBT], and it becomes a problem. It is good luck when your parents understand/accept you” (Nyarungenge, Student, IDI). The isolation and discrimination were further shared in an FGD, “Do you remember what happened to TJ? He ended up leaving town because his mom told church members about him being gay, and they made it a big deal. She was even always trying to find girlfriends so that he would come back to “normal” and stop shaming her” (Nyanza, R1, FGD).

Besides families, othering is pervasive in the larger society as shown through a narration of the dichotomy between a participant’s experience in family and the larger society, “Yes, personally in my family they don't have any problem with it, but in the general community once they learn that you are gay, they will discriminate against you” (Nyanza, R5, FGD). Isolation also occurs within residential communities. For example, “…they [neighbors] even tell the landlord never to allow any of their children to come to my house” (Muhanga, R9, FGD). As another participant added in the same FGD, “We do not admit it publicly [because] they accuse us of wanting to influence their children” (Muhanga, R3, FGD). And as another group member stated, “Well I was expelled from the house simply because they knew I am a lesbian” (Muhanga, R6, FGD). As described, simple acts of daily living, such as living in residential communities, become encumbered because of the othering.

Securing employment was another major area described by participants as impacted by othering. When discussing access to employment, a group conversation unfolded as follows:

“R4: If they know who you are, they will fire you immediately.

R10: If they know who you are before they hire you, they will consider you disqualified.

R6: It is uncomfortable because you will be disqualified regardless of how hardworking you are. Most of the time, you will do all things possible to make them believe that you are not a member of the LGBT community to maintain your job” (Kigali, R4, R10, R6, FGD).

Challenges in staying employed and job promotion because of one’s sexual orientation were also expressed in IDIs, “They [LGBT] do not disclose themselves at work because they know it would have an impact on them” (Kigali, Chef, IDI). Women had even more difficulty in securing employment, and one bisexual woman interviewee shared her experience with being solicited for sex, “I had a job…I was the human resource officer…the challenge I met is that they wanted me to have sex with them to continue working, I refused…so I ended up losing my job” (Kigali, Lawyer, IDI). A key informant concurred with the challenge in securing employment: “The first reason is our culture… their principles are different from our cultural norms, making it even harder for the person willing to provide the job because they are doubtful. Their physical appearance and behaviors make it hard for them to be employed.” (Kigali, IDI, Police Commandant).

### Hampered Services and Capacity to Live as Outcomes of “Othering”

In addition to social isolation, “othering” negatively impacts access to various rights and services, such as education, the capacity to participate in cultural and religious practices, health services, and social media. Participants described health services as a major area being influenced by discrimination. Participants discussed how some LGBT individuals were denied services or referred by healthcare providers to conversion therapy or mental health services. As a participant shared, “Mostly at the hospitals, there are times people take too much time discussing who we are instead of giving us the treatment we want to look for, they even refuse to give us what we want and send us to mental health to first deal with sexuality issues. Since they think it is a sickness” (Muhanga, R1, FGD). Participants noted that most health facilities in Rwanda were faith-based and described the negative influences of religious beliefs on healthcare provision:

“They [faith-based health care] may treat you, but they will give you a bad attitude and make you wait unnecessarily. I have heard of kids who were paraded from provider to provider, including medical students, for them to observe what the cauliflower anal infection looks like. This was done without their consent…. Even if they got the care and treatment, of what quality was it? How do you think they went home feeling? I am sure they still feel traumatized and embarrassed” (Kigali, VIP Sales Associate, IDI).

Discrimination in education was a particular issue discussed among younger LGBT participants. While discrimination in learning institutions was not commonly mentioned, participants reported pockets of stigmatization. It was noted that teachers make negative remarks about LGBT people when a mistake is committed by those suspected to be sexual and gender minorities, “When a teacher sees a mistake made by a member of the LGBT community, they tend to make statements like it is because they are these kinds of people instead of seeing the mistake as a mistake that other straight students also commit, and this is not fair” (Kigali, R7, FGD). In the same FGD, a participant described how their scholarship was withdrawn based on sexual orientation: “I would like to emphasize what my colleague has said about discrimination in schools. I applied for a scholarship, and in the end, even after being approved, the Rwandan coordinators discovered that I was a part of the LGBT community after an investigation, and they canceled my scholarship” (Kigali, R2, FGD). Another participant whose children were reportedly victimized through denial of bursary shared their experience, “…well we were denied many services, they refused to consider my kids for compassion aid because I am a lesbian, so my child became a victim” (Muhanga, R1, FGD).

Social media is also a growing site for othering. Participants widely discussed experiences of othering on social media and how social media is furthering homophobia. As a participant stated, “There is a way you post a video on social media, you see a lot of negative comments from people who do not know us personally. If there is a positive comment, it is mostly from someone who knows us personally. Meaning that people judge us even before they know who we are.” (Kigali, R1, FGD).

Lastly, the ability of LGBT people in Rwanda to participate in their cultural and religious practices is hampered. Participants lamented the ostracization of LGBT people from religious practice. As a participant explained, “Here, no religion accepts what we do because when they find out, they can even chase you out of the church. So, when it comes to religion, it’s not allowed. Personally, I stay at home when everyone goes to church. They are used to it now” (Nyanza, R6, FGD). Another participant explained how they were excommunicated from the church when their sexual orientation was disclosed, “…there is a person who we once talked to. This person reported us to church, and they ended up chasing us away” (Muhanga, R8, FGD). As one pastor explained how the church, through conversion therapy, restores some LGBT people to “God’s original plan,” while those who do not want to change disappear, “I would say, there are those who have disappeared from the church, perhaps they felt rejected, but that was not the intention. Some still come, and some gave testimonies that God had delivered them from homosexuality” (Muhanga, Pastor, KII). Another pastor, who, according to interview notes, seemed uncomfortable with the topic of the interview at the onset and stood up to leave, shared, “First of all according to what you said [about LGBT] that is not in our culture. Second, that is opposite to what the Bible says; as a pastor, I am disappointed to hear that, so I would like to know how those people live and where they are located.” And during the ensuing interview, the pastor endorsed discrimination against LGBT people in the church, “Our mandate is to love and accept them, but acceptance does not mean agreement, which is why tolerance for such behavior within the church is impossible” (Nyanza, Pastor, KII).

## Discussion

The findings from this mixed-methods cross-sectional study in Rwanda indicate that there is a high level of othering and stigmatization of the LGBT community across domains such as housing, employment, healthcare, education, religion, and family within Rwanda, which may be more heightened among transgender-identifying persons. Our study shows that the othering of LGBT people negatively shapes their ability to live authentically and with dignity in the country and serves to isolate them within society.

Research on LGBT issues in Africa, while nascent, is growing and is especially needed given that bigoted perspectives are being reinforced in Africa by Western religious organizations (McKenzie & Dean, 2023). The anti-LGBT efforts in Africa are an extension of the colonial criminalization of same-gender-loving people. African politicians and society often frame the anti-LGBT rhetoric, policies, and movements as a fight against imperialism and “Western values.” However, homophobia and transphobia, as concepts, are rooted in colonial morals and values, and the anti-sodomy laws were introduced during colonialism (Currier & Gogul, 2020; University of Pretoria, 2023). In many traditional African cultures, such as the royal clans of Baganda (the largest ethnic group in Uganda), women were addressed with male titles (Kalende, 2023). Ethnographic evidence of same-gender relationships in pre-colonial Africa is also found among the Azande of Congo, the Beti of Cameroon, the Pangwe of Gabon, and the Nama of Namibia (Kalende, 2023). The colonial expansion of anti-LGBT bigotry is further problematized by its roots in religious indoctrination and Christianity, which itself was imported into Africa during colonialism. During colonialism, traditional conflict resolution mechanisms, such as tribal councils and village courts, were replaced with homophobic and transphobic European penal codes that criminalized same-sex relations (Kalende, 2023). These penal codes are being expanded, which can be seen most recently in the Ugandan legislation that criminalizes same-sex behaviors and, in certain instances, such as non-disclosure of HIV, can be sentenced to death (Nicholls & Princewill, 2023). Research regarding LGBT people’s rights and health must grapple with the colonial introduction of anti-LGBT laws and the current anti-imperialist fights against “Western value,” which can be found in other geographic spaces, such as Eastern Europe, where LGBT human rights are being framed as western imports (Ayoub & Paternotee, 2014; Misovska Kajevska, 2016; Stojanovski et al., 2020).

While perspectives of LGBT rights are split in Rwanda, as our findings show, people acquainted with LGBT are less discriminatory. This finding indicates that social interactions and education campaigns with LGBT people and concepts may serve to improve knowledge and attitudes within society, which can reduce stigma. Sensitivity training with healthcare providers in coastal Kenya found that the training reduced homophobia (van der Elst et al., 2013). Research indicates that training for healthcare professionals about LGBT people and their needs is needed globally (Baiocco et al., 2022; Dijkstra et al., 2015; Keuroghlian et al., 2017). Such training and social education campaigns have been even more impactful in countries with high stigma as they serve not only as an individual-level intervention but also as a social network intervention by shaping subjective norms among persons within social networks (Ajzen, 1991). Social education and sensitivity training with critical players in Rwandan society might be a practical, immediate action to decrease the othering of LGBT people in Rwanda.

Research portrays how social media has been used as a helpful tool for activism and social change. Transnational conversations across and within LGBT non-governmental organizations are flowing regarding advocacy and safeguarding rights (Holzhacker, 2012). However, our results showed that discrimination and othering also occurred online. Transnational solidarity and work have been instrumental in improving attitudes and laws regarding LGBT people globally. However, it also created tension by viewing more financially powerful and backed LGBT organizations as “an outsider,” where anti-West and anti-imperialism backlash rapidly and regularly unfolds (Kahlina, 2015; Paternotte, 2016; Stojanovski et al., 2020). Community practice models would be important considerations in local Rwandan and the more significant African LGBT movement.

Our findings also indicate the importance of incorporating intersectional lenses into LGBT research, given results, both quantitatively and qualitatively, showing that trans-identifying people experience more discrimination. Given the additional marginalization across gender identity, sex, and physical appearance (intersectionality), trans-identifying people in Rwanda experience disproportionate othering and its impacts. Global trans research indicates this to be a widespread phenomenon with significant negative implications for health (Restar et al., 2020; Scheim et al., 2016; Stojanovski et al., 2015, 2019; White Hughto et al., 2015). Intersectionality theory may play a particularly important role in global LGBT research, focusing on how power, its use, and its relationship to the allocation of resources shape lived experiences (Crenshaw, 1989). Given that advantages and disadvantages vary across countries due to different socially stratified identities such as gender identity, ethnicity, sexual orientation, religion, and tribe global LGBT research would greatly benefit from examining the similarities of power structures and norms.

## Limitations

As with any research study, limitations exist. First, the cross-sectional nature of this study limits our ability to rule out reverse causation (e.g., discrimination might shape how many LGBT people folks know). However, for the LGBT survey, identity, and its relationship to discrimination, reserve causation would be more challenging to argue. Given the convenience and respondent-driven sampling methods, the study is also not generalizable outside the research population and the usefulness of the sample size calculation is constrained. In addition, we were missing a variable to create RDS-adjusted estimates, which limited our capacity to correct for unequal sampling probabilities. In addition, nearly half of the sample (46%) was comprised of gay male participants, but we were able to achieve a relatively large sample of bisexual (24%), lesbian (16%), and trans-identifying persons (14%) who are often unrepresented in global LGBT research. Moreover, the large sample sizes for such a study are commendable and offer rigorous insights into LGBT issues in Rwanda. However, additional work with non-conforming, genderqueer, or agender subgroups is particularly important given their limited inclusion within research.

## Conclusion

Cultural, social, and religious norms rooted in “anti-West” and “un-African” viewpoints are the root causes of othering of the LGBT community in Rwanda, with trans and non-confirming identifying people in Rwanda experiencing disproportionate othering. Social education and targeted bias reduction training campaigns with key societal players (e.g., healthcare providers, police) may have essential roles in addressing LGBT othering in Rwanda.

## Data Availability

The data are not publicly available.

## Code Availability

Statistical coding is available upon request.
